# Supplementary material for: The Experience of Apathy in Dementia: A Qualitative Study
Source: Int J Environ Res Public Health. 2021 Mar 23;18(6):3325. doi: 10.3390/ijerph18063325 (PMC8005167; doi:10.3390/ijerph18063325)
Supplement: Supplementary file 1 [file ijerph-18-03325-s001.pdf]

Supplementary material: semi-structured Interview

Demographic information:

1) Age
2) Sex
3) Marital status
4) Previous occupation or profession
5) Duration since diagnosis of dementia

Experience of apathy:

1) What does ‘apathy’ mean to you?
2) Tell me about your first experience of apathy
3) When did it start?
4) Does it happen most of the day, almost every day?
5) What does it feel like?
6) What is a typical day like for you/ your daily routine?
7) How does your experience vary day by day?
8) Anything in particular that makes it worse?
9) Anything in particular that makes it better?
10) Do you have any hobbies or activities that you enjoy?
11) Do you have any hobbies or activities that you no longer feel motivated to engage in?
12) Effect on i) daily activities, ii) mood, iii) relationships (including relationship with partner)
13) Passive or withdrawn?
14) Did you notice lack of or putting less effort into your everyday activities?
15) If an extra effort is needed do you refrain from doing things?
16) Are you less helpful in household chores?
17) Do you feel that in order to carry out your daily activities you need that someone indicates or organises your activities?
18) Do you need a “push” to start doing your daily activities?

19) Do you feel that you have less interest to learn new things? (for example, reading newspapers, books, watching TV)?
20) Do you note less interest to learn about events in your environment, such as family meetings, neighbourhood news?"
21) Are you indifferent or less interested in your personal situation?
22) Are you indifferent to your health condition, working situation, hobbies, or personal care?
23) Do you have a flat mood, with little or no fluctuations?
24) Do you feel indifferent in your emotions, neither happy nor sad about positive or negative events in your environment?
25) How do you deal with it?
26) Do you have any form of support?
27) Your reaction to it?
28) Social circumstances
29) How much does it worry you?
30) Anything else you would like to add?

Funding

No external funding was received for this study.

Author contributions

All authors contributed substantially to the work, as follows. TD and JY conceived of the study and designed methodology. WB and JY conducted interviews with people with dementia and with carers, respectively. WB obtained the interview transcripts and performed the first round of analysis, after which all four authors contributed equally to analysing the data. MC led on the analysis of data from the accompanying carer interviews, which will reported separately. WB wrote the first draft of the paper but all four authors were actively involved in reviewing and editing the manuscript.TD and JY acted as supervisors for WB and MC.
